# Knowledge, attitudes, and awareness of breast self-examination among female university students in three Arab countries: a multi-center cross-sectional study

**DOI:** 10.1186/s12889-025-23932-5

**Published:** 2025-08-27

**Authors:** Aliaa Gamal, Sara Adel Awwad, Abdallfatah Abdallfatah, Ahmed Adel Abdel Azim, Waleed Alhayek, Hagar Elgamal, Sarah Saleh Omar, Noura Waleed Koura, Abdelrahman Sameh Soliman, Abdelazeez Ahmed Masoud, Obai Yousef, Mohamed Abouzid

**Affiliations:** 1 Clinical Pharmacy Department, Faculty of Pharmacy, Deraya University, Minia, Egypt; 2 Faculty of Medicine, Jordan University of Science and Technology, Irbid, Jordan; 3 Faculty of Medicine, October 6 University, Cairo, Egypt; 4 Faculty of medicine, Assiut university, Assiut, Egypt; 5 Faculty of Medicine, Latakia University, Latakia, Syria; 6 Public Administration of Dental Health, Health Affairs Directorate Alexandria, Alexandria, Egypt; 7 Faculty of Medicine and Health Sciences, Aden University, Aden, Yemen; 8 Faulty of Medicine, Kafr El-sheik University, Kafr El-sheik, Egypt; 9 Faulty of Medicine, Alexandria University, Alexandria, Egypt; 10 Faculty of Medicine, Tartous University, Tartous, Syria; 11 Department of Physical Pharmacy and Pharmacokinetics, Faculty of Pharmacy, Poznan University of Medical Sciences, Rokietnicka 3 St, Poznan, 60-806 Poland; 12 Doctoral School, Poznan University of Medical Sciences, Poznan, 60-812 Poland

**Keywords:** Breast Cancer, Knowledge, Awareness, Attitude, University students

## Abstract

**Background:**

Breast Cancer (BC) is a significant leading cause of death among women. With an estimated 2.1 million new cases annually, it represents the most prevalent malignancy affecting women worldwide. The early detection of diseases plays a pivotal role in reducing morbidity and mortality. Therefore, this study aimed to assess the level of knowledge, attitude, and awareness of early BC detection among female university students.

**Methods:**

We conducted a multi-national, multi-center, cross-sectional study among a convenience sample of female undergraduate Arab medical students in three countries. A web-based questionnaire was distributed among the students in Arabic and English.

**Results:**

The total number of participants was 1361 (431 from Egypt (32%), 430 from Jordan (32%), and 500 from Syria (37%)). Almost half of the participants showed poor knowledge regarding both risk factors and clinical features of BC (52.5%). The mean scores for knowledge of early detection measures were 59.9% in Egypt, 69.2% in Jordan, and 75.2% in Syria. Regarding knowledge and attitude toward breast self-examination (BSE), the proportion of students with adequate awareness was 16.4%, 17.4%, and 26.8% for Egypt, Jordan, and Syria, respectively. A significant proportion of students had heard about BSE (72.74%), and more than half knew the recommended frequency (62.12%). However, only 33.15% of students regularly performed the BSE at least once every month. Furthermore, a predominant factor contributing to students’ non-compliance with the BSE was their lack of knowledge regarding the proper technique (41.92%). The most predictive factor for the practice of BSE was a family history of BC, and the least predictive factor was knowledge level. Mammogram was the most commonly recognized breast cancer screening method, among students (82%), and social media was the most common source for acquiring knowledge on BC and BSE (38%).

**Conclusion:**

The study showed a lack of knowledge and awareness among students, highlighting the need for comprehensive educational interventions and training programs within university curricula. These measures are essential for enhancing the understanding of BC and promoting the adoption of BSE practices.

**Supplementary Information:**

The online version contains supplementary material available at 10.1186/s12889-025-23932-5.

## Background

### Breast cancer burden in the Arab world

Breast cancer (BC) ranks as the second most frequently diagnosed malignancy globally and is the leading cancer among women both worldwide and in the Eastern Mediterranean Region (EMR) [1]. It affects approximately 2.1 million women each year, accounting for 25% of all cancers in women [2]. Despite its high incidence, approximately 89% of women diagnosed with BC in Western countries survive five years post-diagnosis, owing to the progress made in early detection and treatment methods [3]. Estimates indicate that approximately one-third of all malignancies can be prevented, and another one-third of malignancies, such as cervical, breast, colorectal, and skin cancers, have a high likelihood of being cured if they are diagnosed at an early stage [4]. Developing countries account for 53% of cancer incidence and 56% of cancer deaths, which suggests a need to review population awareness and screening methods application in developing countries [2]. 

According to the National Cancer Registry Program and Global Cancer Observatory [3], BC remains one of the most prevalent cancers in Egypt, ranking second after hepatocellular carcinoma and accounting for 38.8%.^5,6^ In 2020, the World Health Organization reported an incidence rate of 50.2 cases per 100,000 women in Syria [7]and according to the Al Bairouni Hospital, which treats 60% of Syrian cancer patients, the BC incidence rate accounted for 24% of all cancer cases admitted. According to the most recent statistics from the Jordan Cancer Registry, the most common cancer is BC, accounting for 39.4% of cancers among Jordanian women [8]. 

In Egypt [9, 10]Jordan [11]and Syria [12]most BC cases are present as advanced stages or more aggressive metastatic tumors rather than early stages. This is attributed to limited awareness, barriers to healthcare access, and sociocultural stigmas. Alarmingly, research findings reveal that a significant majority of Egyptian women, approximately 85%, have an insufficient understanding of BC. This highlights the urgent need to improve young women’s knowledge of BC and encourage the early identification of the disease [13, 14]. 

Late identification of BC decreased the 5-year survival rate to 56%, whereas early detection increased the survival rate to 85%, leading to positive outcomes and quality of life for women [15]. World Health Organization (WHO) endorses breast self-examination (BSE) as a valuable technique for reducing BC mortality through early detection, particularly in areas where mammography and routine clinical exams are not readily available. BSE is emphasized for its convenience, safety, and lack of need for specialized equipment, making it particularly suitable for developing countries. Its primary goal is to familiarize women with the normal appearance and feel of their breasts to facilitate the prompt identification of any changes [16, 17]. 

Previous cross-sectional studies have shown that basic cancer knowledge among medical students is poor, fragmented, and unorganized, as well as that there is suboptimal knowledge of cancer screening recommendations [18]. Studies indicate that 85% of Egyptian women lack sufficient understanding of BC, and 83% of Jordanian students demonstrated poor awareness, with 90% unfamiliar with major risk factors [19]. Another study that was conducted in one university in Egypt discovered that regardless of students’ year of enrollment in medical school or their graduation status, a relatively low to moderate level of knowledge about cancer screening was found among the chosen medical students, which may be an obstacle for early cancer detection [20].

### Rationale for focusing on young female undergraduates

This study focuses on female university students, a population often overlooked in cancer prevention strategies despite being at a pivotal developmental stage. Young adulthood is a formative phase in which health-related knowledge and behaviors are established and tend to persist into later life [21, 22]. Therefore, university students are ideal candidates for early health education interventions that can yield long-term benefits. Moreover, 72% of our sample comprised healthcare students who, as future professionals and influencers in their communities, are strategically positioned to disseminate BC knowledge to both peers and at-risk relatives.

Further emphasizing the need for early intervention, studies have shown that BC in Arab countries often presents at younger ages compared to Western nations, with a median diagnosis age of 44.5 years in Arab populations versus 60 years in the United States [23, 24]. This age disparity justifies the need to target awareness and screening behaviors among women in their early 20 s before reaching higher-risk age groups. The concern that these young women may not yet have undergone comprehensive health education is precisely why they were selected.

### Global comparative evidence on breast Cancer knowledge and practices

Knowledge, attitudes, and practices (KAP) deficiencies among young women have been well-documented globally. In China, Zhang and Yu et al.‘s 2022 study found low knowledge (mean score: 9.86/15), with education level being a key predictor of awareness and behavior toward BC [25].

In Egypt, a study found that 72.4% of medical students were knowledgeable about BSE, compared to only 47.6% of non-medical students. Despite this difference, both groups demonstrated generally inadequate attitudes toward BC awareness and prevention [26]. Cameroon’s study by Nde et al.,2015 revealed that although 73.5% had heard of BSE, only 9% knew how to perform it, and a mere 3% practiced it regularly [17]. Similarly, Asmare et al.‘s 2022 study in Ethiopia showed that 56% had adequate knowledge, yet only 45.8% practiced BSE, often due to a lack of structured educational programs [27].

In Saudi Arabia, 77.6% of women had poor knowledge, and 94% did not practice BSE, despite 79.6% having heard of it, highlighting a disconnect between awareness and implementation [28]. Jadhav et al., 2024 in India noted similarly troubling trends: 58% lacked knowledge, 73.8% held unfavorable attitudes, and 89.6% did not practice BSE, with cultural barriers being significant deterrents [29]. In Malaysia, 74% of female health sciences students were knowledgeable, and 99% had positive attitudes, yet only 42% practiced BSE monthly, often hindered by curriculum gaps [30]. UAE data revealed that 91.5% of women believed BC to be curable, 93% understood early detection’s importance, and only 46% practiced BSE [31].

This broader body of research underscores that the deficiencies identified among university students in Egypt, Syria, and Jordan are not unique but rather reflect a pervasive global issue in breast cancer education and preventive practice. Thus, the present study reinforces existing literature and contributes a regional lens to a universal health disparity, highlighting the urgent need for culturally tailored interventions and educational reforms to enhance early detection behaviors among young women.

## Methods

### Study design and setting

We conducted a multi-national, multi-center cross-sectional study among female university students in three countries in the MENA region (Jordan, Egypt, and Syria) between March 1, 2023, and June 1, 2023, using an online self-administered questionnaire.The survey was distributed online using Google Forms and available in English and Arabic. The Strengthening the Reporting of Observational Studies in Epidemiology (STROBE) checklist was followed in the conduct and reporting of the current article [32]. 

### Population and sampling

This study employed convenience sampling to attain the predetermined sample size. The target population was female university students currently enrolled in universities in Syria, Egypt, and Jordan, regardless of their level of study. A convenience sampling method was employed due to logistical and ethical constraints in obtaining official student lists across multiple institutions. The survey link was disseminated via university-specific Facebook groups, official student Telegram channels, and WhatsApp groups moderated by campus representatives from seven MENA universities: Deraya University, October 6 University, Alexandria University, and Kafr ElSheikh University in Egypt; Jordan University of Science and Technology in Jordan; and Tartous University and Tishreen University in Syria. These platforms were chosen for their widespread use among regional university students. Participation was entirely voluntary and anonymous.

The sample size for each country was determined individually using OpenEpi software version [33] 3.01, based on a 95% confidence level, a 5% margin of error, and a predicted response distribution of 50%, a commonly used conservative estimate in cross-sectional surveys when the actual prevalence is unknown, as it provides the maximum sample size required and ensures sufficient statistical power [34]. The calculated sample size for each country was 384 participants.

### Questionnaire development and validation

An online questionnaire was created in English after thoroughly evaluating relevant literature and studies conducted in countries such as Egypt [35]Syria [36]Ghana [37]and Pakistan [38]. The BC Awareness Measure (Breast CAM), created in 2009 by Cancer Research UK, King’s College London, and University College London, was also assessed. The questionnaire was administered to an oncologist to check the accuracy of the questions. The initial version of the questionnaire was based on the Breast Cancer Awareness Measure (Breast CAM) and modified to fit the local context. An oncologist and a public health expert assessed content validity. The definitions of key terms were as follows:

*Knowledge*: Refers to the understanding of BC, including risk factors, clinical features, and early detection methods, measured by correct responses to questionnaire points. 

*Awareness*: Is the recognition of BC as a health issue and their familiarity with screening practices such as breast self-examination (BSE) and mammography, assessed through questions about prior exposure to these concepts. 

*Attitude*: Reflects the perceptions, confidence, and willingness to engage in BC early detection practices, evaluated via responses and questions about motivations or barriers to performing BSE

The questionnaire was translated into Arabic by two translators proficient in both Arabic and English, and the Arabic version was subsequently translated back into English by two other bilingual individuals. A separate team of two bilingual professionals performed back-translation into English. Discrepancies between the original and back-translated versions were resolved through discussion in a panel involving the translators and three bilingual medical researchers, ensuring conceptual equivalence, cultural appropriateness, and clarity. In addition, we conducted a pilot sampling for both the Arabic and English versions of the questionnaire, obtaining 20 responses. These responses were not included in the analysis but were used to evaluate Cronbach’s alpha for each questionnaire domain. The calculated Cronbach’s alpha coefficient for the pilot study was 0.84, whilst for all responses was 0.835, indicating good internal cosistency.

The online survey form was designed with mandatory fields to ensure data completeness and accuracy, requiring participants to answer all questions before submission. As a result, there were no missing responses, and no missing data imputation was necessary.

Therefore, the questionnaire was divided into three sections:

- The first section focused on sociodemographic characteristics such as age, academic field, academic year, marital status, mother’s education, and place of residency.
- The second section consisted of 3 questions on awareness of BC. It measured the knowledge of BC risk factors and the knowledge of common clinical features of BC. The third question was about the sources of information on BC.
- The third section focused on early detection methods. It was subdivided into two sub-sections, the first on the general knowledge of early detection methods such as mammography, and the second on attitude and specific knowledge of BSE.

The answer to each question included Yes/No/Don’t know, a 5-point Likert scale ranging from 1 (strongly disagree) to 5 (strongly agree), and multiple-choice questions. For each correct answer, the participant received 1 point; otherwise, they received zero. (Supplementary file 1, Questionnaire)

### Ethical considerations

The study was conducted in accordance with the Declaration of Helsinki and the specific regulatory requirements of each participating country. In Egypt, ethical approval was obtained from the Ethics Review Committee of the Faculty of Pharmacy, Deraya University (Minia). In Syria, approval was granted by the Ethics Committee of the Cancer Research Centre, Tishreen University (Lattakia). In Jordan, the study’s non-interventional, attitude-based design falls under the exemption provisions of the Jordanian Clinical Trials Law No. 10 of 2001 and the Ministry of Health Guidelines for Research Involving Human Subjects, so no formal Institutional Review Board review was required. All participants were informed about the study’s objectives, assured of confidentiality, and provided written informed consent before completing the anonymous questionnaire.

### Data processing and analysis

All statistical analyses included in this paper were carried out using Jamovi software version 2.2.5. The demographic characteristics of the studied sample and their responses to each questionnaire item were reported using frequency tables in the descriptive results section. Participants’ knowledge of BC risk factors and clinical features was scored, and the mean knowledge score was used as the cutoff to categorize respondents into “good” and “poor” knowledge groups. The decision to use the mean was based on the approximately normal distribution of the knowledge scores, verified through the Shapiro–Wilk test, skewness, kurtosis, and visual inspection of histograms and Q-Q plots [39, 40]. 

In addition, binomial logistic regression was used to determine predictors of having ever performed BSE, such as being a healthcare-related field student, having a family history of BC, and having a good knowledge level. These were selected based on their statistical significance (*p* < 0.05) in bivariate analyses. The outcome variable— “ever performed BSE”—was chosen because it reflects a real health behavior, which was central to our study objective. The model was checked for multicollinearity using the Variance Inflation Factor (VIF), with all values below 5. The odds ratio (OR) with a 95% confidence interval (CI) was calculated to assess the degree of association between dependent and independent variables. Confidence and the participant’s determination to visit a doctor for a check-up were also studied using binomial logistic regression. A *p*-value ≤ 0.05 was considered statistically significant in all the analyses included.

## Results

### Sociodemographic characteristics and family history

The study included 1361 female students, with 431 (32%) from Egypt, 430 (32%) from Jordan, and 500 (37%) from Syria. The mean age of the participants was 22 years (SD = 3.60). Most participants, 977 (72%), were in healthcare-related fields. 250 (18%) of participants reported a family history of BC. Table 1.

**Table 1:** Socio-demographic characteristics of the participants

	Frequency (*n*)	Percentage (%)
Age	22.0 (3.60)*	
Academic field		
Healthcare related fields	977	71.79
non-healthcare related fields	384	28.21
Academic year		
First year	162	11.9
Second year	193	14.18
Third year	299	21.97
Fourth year	337	24.76
Fifth year	206	15.14
Sixth year	41	3.01
Internship	123	9.04
Marital status		
Single	1181	86.77
Engaged	77	5.66
Married	52	3.82
I prefer not to say	51	3.75
Mother’s education		
Less than high school	221	16.24
High school diploma	378	27.77
Bachelor’s degree	652	47.91
Master’s degree	74	5.44
Doctorate	36	2.65
Place of residency		
Egypt	431	31.67
Jordan	430	31.59
Syria	500	36.74
Family History of breast cancer		
Yes	250	18.37
No	1111	81.63

*Mean (SD)

### Knowledge of students about the risk factors for BC

The study found that most participants had a good understanding of risk factors associated with tumor history, with 74% acknowledging benign tumors as a risk factor for breast cancer. Personal history of breast cancer was the most well-recognized history-related risk factor. 1,245 (92%), while a family history could increase the risk. However, oral contraceptives and postmenopausal estrogen usage were not recognized as risk factors. Most participants recognized genetic factors well, but 79% thought breast cancer was infectious or did not know. (Supplementary Table 2)

### Knowledge about the common clinical features of BC

Our study found that 91.03% of students correctly associated breast cancer (BC) with a breast lump, 82% believed a lump under the armpit was a symptom, and 76% believed changes in breast shape and size were BC manifestations. Table 2.

**Table 2:** Knowledge of common clinical features related to breast cancer

Common Clinical feature		Overall	Place of residency		
			*N* (%)		
		*N* (%)	Egypt	Jordan	Syria
A lump or thickening in the breast	True	1,239 (1%)	382 (88.63)	390 (90.70)	467 (93.40)
	False	57 (4.2%)	22 (5.10)	19 (4.42)	17 (3.40)
	Don’t know	66 (4.8%)	28 (6.50)	22 (5.12)	16 (3.20)
A lump or thickening under the armpit	True	1,118 (82%)	337 (78.19)	351(81.63)	431(86.20)
	False	120 (8.8%)	48 (11.14)	43(10.00)	29 (5.80)
	Don’t know	123 (9.0%)	47 (10.90)	37 (8.60)	40 (8.00)
A change in pigmentation	True	1,015 (75%)	287 (66.59)	342 (79.53)	387 (77.40)
	False	124 (9.1%)	57 (13.23)	25 (5.81)	43 (8.60)
	Don’t know	222 (16%)	88 (20.42)	64 (14.88)	70 (14.00)
A bloody nipple discharge (other than breast milk)	True	978 (72%)	298 (69.14)	326 (75.81)	326 (65.20)
	False	121 (8.9%)	50(11.60)	28(6.51)	28(5.60)
	Don’t know	262 (19%)	84(19.49)	77(17.91)	77(15.40)
A change in nipple position	True	806 (59%)	238(55.22)	287(66.74)	282(56.40)
	False	207 (15%)	86(19.95)	42(9.77)	80(16.00)
	Don’t know	348 (26%)	108(25.06)	102(23.72)	138(27.60)
A change in the shape of the breast or the nipple (e.g. nipple retraction)	True	1,038 (76%)	317(73.55)	338(78.60)	384(76.80)
	False	115 (8.4%)	43(9.98)	32(7.44)	41(8.20)
	Don’t know	208 (15%)	72(16.71)	61(14.19)	75(15.00)
A change in size of the breast or the nipple	True	1,066 (78%)	324(75.17)	351(81.63)	393(78.60)
	False	120 (8.8%)	52(12.06)	29(6.74)	39(7.80)
	Don’t know	175 (13%)	56(12.99)	51(11.86)	68(13.60)
A breast asymmetry	True	938 (69%)	281(65.20)	286(66.51)	372(74.40)
	False	217 (16%)	81(18.79)	67(15.58)	70(14.00)
	Don’t know	206 (15%)	70(16.24)	78(18.13)	58(11.60)
A scaling or thickening of the nipple or breast skin	True	862 (63%)	270(62.65)	279(64.88)	314(62.80)
	False	145 (11%)	51(11.83)	38(8.84)	57(11.40)
	Don’t know	354 (26%)	111(25.75)	114(26.51)	129(25.80)
A redness of the nipple or breast skin	True	715 (53%)	229(53.13)	247(57.44)	240(48.00)
	False	263 (19%)	93 (21.58)	62(14.42)	109(21.80)
	Don’t know	383 (28%)	110 (25.52)	122(28.37)	151(30.20)
A skin rash on the breast	True	555 (41%)	188(43.62)	204(47.67)	164(32.80)
	False	387 (28%)	121(28.07)	94(21.86)	173(34.60)
	Don’t know	419 (31%)	123(28.54)	133(30.93)	163(32.60)
A pain in one of the breasts or the armpit	True	961 (71%)	307(71.23)	308(71.63)	347(69.40)
	False	223 (16%)	121(28.07)	94(21.86)	173(34.60)
	Don’t know	177 (13%)	123(28.54)	133(32.60)	163(32.60)
	Place of residency	*N*	Mean	*P* value	
Knowledge of common clinical features of BC percentage	Egypt	431	66.8	0.005	
	Jordan	430	71.6		
	Syria	500	68.9		

### Knowledge of early detection measures

The study revealed that 44% of participants were unaware of ultrasound imaging as a detection method of BC and 32% of BSE, while 82% correctly identified mammography, with students from Syria showing the highest awareness **(**Table 3**).**

**Table 3:** Knowledge of early detection measures

The following measures are used for the early detection of breast cancer:			*N* = 1,361	
Breast self-examination	True		914 (67.16)	
	False		141 (10.36)	
	Don’t know		306 (22.48)	
Mammogram	True		1,119 (82.22)	
	False		41 (3.01)	
	Don’t know		201 (14.77)	
Ultrasound	True		763 (56.06)	
	False		153 (11.24)	
	Don’t know		445 (32.70)	
	Place of residency	*N*	Mean	*P* value
Knowledge of early detection methods percentage	Egypt	431	59.9	< 0.001 ^†^
	Jordan	430	69.2	
	Syria	500	75.2	

Data are presented as *n* (%)

^†^*P* value between all three countries

### Knowledge and practice of breast self-examination

Of 990 participants aware of BSE, 615(62%) recognized that it should be practiced monthly, but only 127(33%) do it regularly. However, 52% had never engaged in BSE, and the most frequent justification for that was that they did not know how to do BSE (42%) (Table 4**).**

**Table 4:** Knowledge and practice of breast Self-Examination (BSE)

Variable	Egypt	Jordan	Syria
*Have you heard before about BSE?			
Yes	230 (53.36%)	320 (74.42%)	440 (88%)
No	201 (46.64%)	110 (25.58%)	60 (12%)
^a^How often do you think BSE should be performed?			
Daily	8 (3.48%)	4 (1.25%)	6 (1.36%)
Weekly	16 (6.96%)	29 (9.06%)	35 (7.95%)
Monthly	157 (68.26%)	181 (56.56%)	277 (62.95%)
Yearly	33 (14.35%)	82 (25.63%)	96 (21.82%)
Don’t Know	16 (6.96%)	24 (7.50%)	26 (5.91%)
^b^When do you think is the right time for a woman to perform BSE?			
Anytime	39 (16.92%)	62 (19.38%)	78 (17.73%)
Before menstruation	7 (3.04%)	16 (5.00%)	7 (1.59%)
Middle of menstruation (day 3–5)	19 (8.26%)	19 (5.94%)	75 (17.05%)
Any day during menstruation	3 (1.30%)	5 (1.56%)	4 (0.91%)
After menstruation	103 (44.78%)	98 (30.63%)	173 (39.32%)
Don’t Know	59 (25.65%)	120 (37.50%)	103 (23.41%)
^c^Have you ever performed BSE?			
Yes	98 (42.61%)	98 (30.63%)	187 (42.50%)
No	120 (52.17%)	194 (60.63%)	206 (46.82%)
Don’t Know	12 (5.22%)	28 (8.75%)	47 (10.68%)
**If not, why?			
Not sure of its ability to detect BC	10 (8.33%)	26 (13.40%)	24 (11.65%)
Fear of positive finding	3 (2.50%)	9 (4.64%)	6 (2.91%)
Don’t Know how	42 (35.00%)	72 (37.11%)	104 (50.49%)
Forgetting	27 (22.50%)	33 (17.01%)	30 (14.56%)
Not interested	38 (31.67%)	54 (27.84%)	42 (20.39%)
^***^If yes, how often do you check your breast?			
At least once every month	41 (41.84%)	30 (30.61%)	56 (29.95%)
At least once every 6 months	26 (26.53%)	26 (26.53%)	60 (32.09%)
At least once a year	2 (2.04%)	9 (9.18%)	22 (11.76%)
Rarely	29 (29.59%)	33 (33.67%)	49 (26.20%)
^†^ How confident are you that you would notice a change in your breast?			
Not confident at all	15 (15.31%)	9 (9.18%)	7 (3.74%)
Not very confident	37 (37.76%)	33 (33.67%)	49 (26.20%)
Fairly confident	27 (27.55%)	45 (45.92%)	111 (59.36%)
Very confident	19 (19.39%)	11 (11.22%)	20 (10.70%)
^†*^ Would you go see a doctor to check your breast if you were very confident about detecting a change in your breast?			
Yes	88 (89.80%)	88 (89.80%)	176 (94.12%)
No	10 (10.20%)	10 (10.20%)	11 (5.88%)
^†**^ Would you go to see a doctor to check your breast if you were fairly confident about detecting a change in your breast?			
Yes	70 (71.43%)	65 (66.33%)	120 (64.17%)
No	28 (28.57%)	33 (33.67%)	67 (35.83%)

^a,b,c^ *Egypt N = 230 Jordan N = 320 Syria = 440*

^*^ *Egypt N = 431 Jordan N = 430 Syria N = 500*

^**^ *Egypt N = 120 Jordan N = 194 Syria = 206*

^***^ *Egypt N = 98 Jordan N = 98 Syria = 187*

^†, †*, †**^ *Egypt N = 98 Jordan N = 98 Syria = 187*

Interestingly, the most substantial factor in not performing a clinical examination in a specialist medical center was financial concern, as 71% of Egyptians, 75% of Jordanians, and 82% of Syrians did not want to spend money on a test they were unsure they necessarily needed. Followed by not wanting to panic the family by informing them about the desire to have a clinical examination (25–33%). Despite the stereotypes of Arab societies, social shyness was the least influencing factor that may affect one’s decision to visit a doctor in Egypt (13%) and Syria (12%). In contrast, the least influencing factor in Jordan was fear of a positive result (15%) (Table 5**).**

**Table 5:** Factors influencing hesitation or refrainment from performing clinical breast examination

	Place of residency	Strongly Disagree	Disagree	Neutral	Agree	Strongly Agree
Fear of positive result	Egypt	33(33.67)	28(28.57)	15(15.31)	17(17.35)	5(5.10)
	Jordan	34(34.69)	23(23.47)	26(26.53)	13(13.27)	2(2.04)
	Syria	47(25.13)	69(36.90)	35(18.72)	35(18.72)	1(0.53)
Social shyness	Egypt	32(32.65)	31(31.63)	22(22.45)	12(12.24)	1(1.02)
	Jordan	38(38.78)	30(31%)	8(8.16)	19(19.39)	3(3.06)
	Syria	65(34.76)	82(43.85)	18(9.63)	21(11.23)	1(0.53)
Don’t want to panic your family	Egypt	25(25.51)	17(17.35)	24(24.49)	26(26.53)	6(6.12)
	Jordan	30(30.61)	20(20.41)	17(17.35)	23(23.47)	8(8.16)
	Syria	37(19.79)	70(37.43)	33(17.65)	41(21.93)	6(3.21)
Don’t want to spend money on a test that you are not sure is necessary for you	Egypt	2(2.04)	3(3.06)	14(14.29)	35(35.71)	44(44.90)
	Jordan	4(4.08)	2(2.04)	19(19.39)	29(29.59)	44(44.90)
	Syria	2(1.07)	13(6.95)	20(10.70)	72(38.50)	80(42.78)

*Total participants - Egypt N = 98, Jordan N= 98, Syria N = 187*

### Sources of information on BC

The study found that social media was the most common source of knowledge, while television was the least used source (3.7%) (Supplementary Table 1 and Supplementary Fig. 1).

### Correlations and associations

The performed bivariate analysis acted as an exploratory analysis to guide the selection of variables for the logistic regression depends on the results of multiple logistic regression. In Syria, individuals from healthcare-related fields and those from non-healthcare-related fields were statistically different regarding their knowledge of BC’s risk factors, common clinical features, early detection methods, and attitudes toward BSE. The same applies to Egypt and Jordan, with the attitude toward BSE being an exception (*p* = 0.381 for Egypt and *p* = 0.185 for Jordan) (Table 6**).** Upon performing a binary logistic regression, a positive significant association was found between having a family history of BC and practicing BSE in all countries. However, knowledge levels did not predict BSE engagement. Additionally, Logistic regression analysis revealed that having good knowledge could not predict whether a student had ever practiced BSE in any of the three countries **(**Table 7**).**

**Table 6:** Analysis of knowledge level across academic fields

	Academic Field	*N*	Knowledge of BC’s risk factors	Knowledge of BC’s common clinical features	Knowledge of BC’s early detection methods	Knowledge and attitude towards BSE	Total score
Egypt	Health care related	347	59.6	68.5	62.2	18.1	44.9
	Non-health care related	84	44.6	59.8	50.4	9.42	33.9
*P* value			< 0.001	< 0.001	0.004	0.381	< 0.001
Jordan	Health care related	328	57.4	74.6	71.3	18.2	45.9
	Non-health care related	102	46.1	62.3	62.4	14.5	37.6
*P* value			< 0.001	< 0.001	0.029	0.185	< 0.001
Syria	Health care related	302	58.8	74.3	80.5	28.7	51.1
	Non-health care related	198	43.9	60.8	67.2	23.8	40.6
*P* value			< 0.001	< 0.001	< 0.001	0.004	< 0.001

Data are reported as %

*BC* Breast Cancer

*BSE* Breast self-examination

**Table 7:** Binary logistic regression

Country	Variables	Practice of Breast self-examination		Odds Ratio	95% confidence interval		*P* value
		Yes	No		Lower	Upper	
Egypt	Academic field Health care related field Non health care related field	89(25.6) 9(10.7)	258(74.4) 75(89.3)	0.348	0.167	0.723	0.005
	Family History of Breast Cancer Yes No	26(34.2) 72(20.3)	50(65.8) 283(79.7)	2.044	0.196	0.330	0.009
	Knowledge level Good Poor	83(19.3) 15(3.5)	254(58.9) 79(18.3)	0.581	0.317	1.064	0.079
Jordan	Academic field Health care related field Non health care related field	80(24.4) 18(17.6)	258(74.4) 75(89.3)	0.664	0.376	1.172	0.158
	Family History of Breast Cancer Yes No	21(33.9) 77(20.9)	41(66.1) 291(79.1)	1.936	1.081	3.467	0.026
	Knowledge level Good Poor	84(19.5) 14(3.3)	267(62.1) 65(15.1)	0.685	0.366	1.282	0.237
Syria	Academic field Health care related field Non health care related field	80(24.4) 18(17.6)	258(74.4) 75(89.3)	0.804	0.554	1.168	0.253
	Family History of Breast Cancer Yes No	52(46.4) 135(34.8)	60(53.6) 253(65.2)	0.616	0.402	0.943	0.026
	Knowledge level Good Poor	145(32.2) 42(9.4)	200(44.5) 62(13.8)	0.934	0.598	1.460	0.766

Data are presented as *n* (%)

## Discussion

Our survey revealed that female Jordanian, Egyptian, and Syrian university students were ignorant about BC knowledge and BSE practice. This is the first regional research that looks at understanding the knowledge, attitudes, and awareness of early BC detection among female university students in three Arab nations, which assists in closing this knowledge gap.

In Arab nations, BC is the most prevalent type of cancer among women [38]. According to a previous study, BC manifests at a younger age in Arab countries than in Western countries [41]. Early identification through regular screening activities, such as BSE, clinical breast examination (CBE), and mammography, significantly increases the chance that treatment will be effective and reduce mortality [42, 43]. However, several variables, including a woman’s discomfort and reluctance to reveal their breast to a doctor, social stigma, and concern that a diagnosis may affect relationships with relatives and marriage chances, affect participation in breast screening and early diagnosis [44]. As a result, Arab women are frequently diagnosed with BC at advanced stages [45]. The majority of Arab nations practice opportunistic BC screening, which means that women who take part in screening events are either self-motivated or referred by a doctor; there is no centralized invitation or follow-up mechanism [38].

Our study found that the most common source of participant knowledge about BC was social media, followed by healthcare professionals. These findings were similar to a study on female university students in UAE [3]. This can be explained by the fact that technology is currently utilized the most. Additionally, the individuals in our sample were all university students who tend to be tech-adept; however, our study revealed that they still have inadequate knowledge, which could be explained by forgetfulness or a lack of understanding of how to do BSE properly. Our analysis showed that the most frequently identified risk factor was a family history of BC(91%), followed by genetic factors (89%), which was also in agreement with a study that targeted female medical students in Saudi Arabia (83.6%) [46]. Another study conducted in Jordan among female community pharmacists had similar agreement on family history of BC(97.6%) and genetic factors (93.2%) being the most identified risk factors. The major sign and symptom identified was a lump or thickening in the breast (91%), consistent with previous findings in Egypt and UAE [3, 35]. On the other hand, lump or thickening in the breast was the second identified risk factor in a study that was conducted in Saudi Arabia, as the first one was discharged or bleeding from the nipple [46].

Most students in the three countries of our study identified mammograms (82%) as early detection for BC, followed by BSE (67%) and then ultrasound (56%). Similarly, a study carried out among future health professionals in Ghana showed that mammogram (71.1%) was the most common screening method they were aware of, followed by BSE (69.8%) [36]. However, another study that studied awareness of BC screening among Arab women in Qatar reported that the awareness of BSE (28.9%) was higher than mammograms (26.4%)^47^.

The current study showed that university female students in Syria had the highest percentage who had heard about BSE (88%), followed by Jordan (74%) and Egypt (53%). Another study conducted in Egypt showed higher awareness among students who heard about BSE (63.4%) than our study [35]. On the other hand, our findings showed higher results than previous studies in Syria and Jordan that reported (86%) and (67%) regarding the awareness of BSE practice [36, 48]. We may relate to the fact that our study was multi-national, comparing students from more than one university, and we included more students compared with the Syrian study. Furthermore, the Jordanian study was conducted in 2002, and of course, more knowledge would be gained due to increased technology use and easier access to information. Over (45%) of Egyptian participants knew the right timing, which is a high percentage compared with a previous study in Egypt that reported only (8.8%) of students who knew the appropriate time to perform BSE [35]. Even Syria and Jordan scored (39%) and (31%) respectively, which is higher in comparison with a previous Egyptian study. This is because most participants’ knowledge is derived through social media and technology, and it is simple to obtain any information required in your location with no effort.

More than half of the participants in each country had never performed BSE; the most common reason was they did not know how. In order to overcome and limit these obstacles, future educational programs should emphasize that BC may be curable if caught early. Enhancement, extension, and accessibility of BC Screening to encompass female populations might further increase early detection and lessen obstacles to early presentation. To get the best outcome, this calls for a significant amount of funding in addition to government support and guidance from professionals in the medical area.

Another concerning reason was that they were not interested in performing BSE, with Egypt in the top (32%), followed by Jordan (28%) and Syria (20%). These findings were consistent with earlier research performed in Jordan and the UAE, which found that most participants do not perform BSE because they do not know how (32.4%), as in UAE, or believe it is not necessary, as in Jordan (32.2%) [3, 49]. Based on that, increased awareness-raising efforts should be made. Our binary logistic regression revealed a significant association between performing BSE in Egypt and having a healthcare background. A survey conducted into the use of breast self-examination among Jordanian students and medical professionals revealed that 856 (90.49%) of the participants had low BSE practice, which can be explained by disparities in the countries’ educational systems and a lack of knowledge about breast cancer [49].

Our current study showed that the highest percentages of participants performed BSE at least once every month, with Egypt being the first (42%), followed by Jordan (31%) and Syria (30%). This finding is supported by a study conducted among women in Wolaita Sodo, Ethiopia, which reported that the most common BSE practice is once every month (45%)^50^. Another study showed that more than half did not know the appropriate frequency to practice BSE (55.6%). Once every month is their second most identified answer among female students at Addis Ababa University, Ethiopia [51].

The current study suggested several reasons why the participants refrained from performing a clinical examination of their breasts or hesitated about the idea. Fear of a positive result was the most common answer in Egypt (34%) and the second most common in Jordan (35%) and Syria (25%). Social shyness was the most identified reason in Jordan (39%) and Syria (35%). As a result, educating women about the potentially life-saving advantages of BSE practice is crucial. Additionally, with government assistance, the accessibility of screening procedures should be increased.

The diversity in educational facilities, availability of resources, faculty instructors, and national health education system all contributed on how breast cancer awareness is taught and viewed. For instance, a research paper that studied barriers and facilitators towards BC Screening in the Arab World showed that Jordanian health care providers were found to have inadequate knowledge of breast cancer screening^47 52^. On the other hand Syria had a conflict-related crisis which impacted both funding and limited the capacity of the health care services.The health care system also faced destruction in their institutions, executions of healthcare providers, and a severe lack of pharmaceuticals and medical equipment, which led to a decrease quality of life. Furthermore, about half of Syria’s physicians fled outside the country, leading to a substantial drop in educational training [52].

Egypt experiences various challenges despite having a long record of academic achievements and many universities. These include classroom overcapacity, limited funds in public institutions, and disparities between urban and rural areas. Furthermore, there is a lack of adequate distribution of the screening centers which led to limited access to breast cancer screening facilities; moreover, lack of time was considered another barrier as they viewed self-care as a low priority [43].

Students’ knowledge is also influenced by their academic level and field of study. Those in higher academic years or pursuing health-related fields such as medicine, nursing, or pharmacy are more likely to have received breast cancer education or have sufficient prior background through coursework or clinical training. However, Students in non-health areas or their early years of study may have had limited exposure to such content despite their national or technology access.

This highlights how important it is that the government carry out a comprehensive and in-depth education program, increase funding for education, engage a larger portion of the population to include all women, and establish proactive, successful educational programs to give students pursuing health professions the necessary knowledge and awareness about the burden of diseases, including BC cancer. However, Since these nations are developing nations, the largest hurdle is money. For instance, Jordan’s socioeconomic status leads to significant disparities in health care coverage. The percentage of Jordanians without health insurance is around 65%; the numbers are lower for those without jobs, those living in rural regions, and refugees. Therapy may be delayed as a result of these differences. Higher incidences of advanced-stage cancer upon diagnosis and higher cancer patient fatality rates are linked to uninsured status.

Socioeconomic and geographic disparities significantly affect access to healthcare in the Arab region, particularly in rural areas. Individuals residing in rural communities often face long travel distances and high transportation costs, contributing to lower healthcare service utilization and higher physician turnover [53, 54]. Hammad et al. investigated how financial constraints affected Jordanians’ access to cancer care and identified critical gaps in existing financial aid programs, particularly concerning the coverage of supportive care services, transportation, and treatment expenses [55]. Similarly, an evaluation of the Goodwill Cancer Care Fund—a national initiative supporting cancer patients—revealed that although many patients benefitted from financial assistance, substantial coverage and accessibility gaps persist [56].

Additional early detection and screening barriers are tied to insurance status and healthcare financing. Donnelly et al. study reported that cost and lack of insurance coverage were among the primary obstacles to breast cancer screening in Jordan [43]. While Jordanians with public insurance receive medications at no cost through governmental clinics, those with private insurance may face variable copayments depending on the terms of their policy. Furthermore, non-Jordanians and uninsured Jordanians must pay out-of-pocket or rely on humanitarian organizations such as UNRWA and UNHCR [57]. These financial challenges are particularly burdensome for refugees and low-income patients, who may struggle not only with affording medications and diagnostics but also with ancillary needs such as travel, temporary accommodation, and sustenance during treatment. These non-medical costs compound the burden of cancer care and underscore the need for comprehensive support systems that address both direct and indirect barriers to treatment.

Our findings align with recent regional studies, particularly Qtaishat et al. [58]which identified low BC knowledge and screening uptake among Arab women, with only 53.4% recognizing ≥ 5 BC symptoms and 72% practicing BSE. Notably, their study highlighted “lack of breast issues” (26.8%) and “fear of results” (35.5%) as key barriers to screening, mirroring our participants’ reluctance due to social shyness (39% in Jordan) and fear (34% in Egypt). However, while their cohort relied heavily on healthcare workers (42.5%) for BSE guidance, our university students predominantly used social media, suggesting generational or educational differences in information-seeking behaviors. These parallels underscore the pervasive cultural and structural challenges across the MENA region, reinforcing the need for standardized, youth-targeted awareness programs that address both knowledge gaps and psychosocial barriers.

## Strength

This is the first multi-center cross-sectional study conducted in three Arab countries (Egypt, Jordan, and Syria) to assess BC knowledge and screening practices among female university students. This approach allows for a more comprehensive understanding of BC awareness and screening behaviors among young women in the Eastern Mediterranean region.

Researchers can identify similarities and differences in knowledge and practices by including multiple countries in the study, enabling them to draw more robust conclusions and develop targeted strategies for each country.

Additionally, focusing on female university students is an important strength as it targets a population with relatively high levels of education and access to information. University students can act as potential change agents and influence awareness and practices within their communities and social networks. Understanding this demographic’s knowledge and screening practices is vital for designing effective interventions and educational campaigns to promote early detection and reduce BC mortality.

## Limitations

This study was based on cross-sectional data that might have restrictions in interpreting any causative relationship as it relied on the accuracy and honesty of the participants’ responses to obtain information. Moreover, convenience sampling was the only available method for achieving the calculated sample size; random or cluster sampling would have been a better approach to decrease selection bias. Convenience can lead to biases, such as a higher likelihood of participation among students who have internet access and often check social media platforms and university networks. These students may differ in lifestyle, cultural background, and education. Future studies could use The Checklist for Reporting Results of Internet E-Surveys (CHERRIES) to limit the bias of the online surveys [59].

Most participants were in health-related fields, which increased their chances of giving the correct answers compared to non-health-related fields. Additionally, most students were in their last academic years, providing them the advantage of being exposed to this topic during their coursework or awareness campaigns in their early years of enrollment. Furthermore, the specific demographic under study consisted of female university students, rendering the findings non-generalizable to the broader population. In addition, this study used a self-administered questionnaire and targeted females with high levels of education who could access the Internet. Consequently, the results of this study cannot be generalized to all females in the community, such as illiterate populations in rural areas with no Internet access. We could not calculate a traditional response rate because the survey link was distributed via open social media and messaging channels.

Finally, the post‑hoc power analyses reveal low statistical power—particularly in Jordan (≈ 23%) and Syria (≈ 6%)—suggesting that moderate associations between knowledge level and BSE practice may have gone undetected. Consequently, the non‑significant odds ratios (ORs 0.58–0.93, all *p* > 0.05) should be interpreted with caution. Future studies should increase sample sizes—especially within the “poor knowledge” stratum—or pool data across centers to achieve at least 80% power. Additionally, employing stratified or random sampling techniques, ideally through partnerships with participating institutions, and combining online with in‑person recruitment methods would improve representativeness and minimize selection bias.

## Conclusion

This study showed a lack of knowledge of BC, and only a few students practice BSE monthly. This has a negative impact on the early detection of BC and increases the burden in such limited-resource countries as Egypt, Jordan, and Syria.

## Recommendations

There is a crucial need for appropriate educational interventions to raise awareness about BC and BSE among female students at the universities of Egypt, Jordan, and Syria, such as electives, free BSE training courses, and awareness campaigns.

## Supplementary Information

Supplementary Material 1.

Supplementary Material 2.

## Data Availability

Data is provided within the manuscript or supplementary information files.

## References

[1] Ibrahim AS, Khaled HM, Mikhail NN, Baraka H, Kamel H. Cancer incidence in egypt: results of the National Population-Based Cancer registry program. J Cancer Epidemiol. 2014;2014:e437971. 10.1155/2014/437971. 10.1155/2014/437971 PMC4189936 25328522

[2] Torre LA, Bray F, Siegel RL, Ferlay J, Lortet-Tieulent J, Jemal A. Global cancer statistics, 2012. CA Cancer J Clin. 2015;65(2):87–108. 10.3322/caac.21262. 25651787 10.3322/caac.21262

[3] Rahman SA, Al–Marzouki A, Otim M, Khayat NEHK, Yousef R, Rahman P. Awareness about breast cancer and breast self-examination among female students at the university of sharjah: a cross-sectional study. Asian Pac J Cancer Prev. 2019;20(6):1901. 31244316 10.31557/APJCP.2019.20.6.1901 PMC7021607

[4] Noreen M, Murad S, Furqan M, Sultan A, Bloodsworth P. Knowledge and awareness about breast cancer and its early symptoms among medical and non-medical students of southern Punjab, Pakistan. *Asian Pac J Cancer Prev*. 2015;16(3). https://ora.ox.ac.uk/objects/uuid:17a2f0d1-586a-4466-9909-ec1fc965ddd7Acc. 21 Jul 2023. 10.7314/apjcp.2015.16.3.979 25735392

[5] Azim HA, Elghazawy H, Ghazy RM, et al. Clinicopathologic Features of Breast Cancer in Egypt—Contemporary Profile and Future Needs: A Systematic Review and Meta-Analysis. JCO Glob Oncol. 2023;(9):e2200387. 10.1200/GO.22.00387. 10.1200/GO.22.00387 PMC10497263 36888929

[6] Tawfik E, Ghallab E, Moustafa A. A nurse versus a chatbot–the effect of an empowerment program on chemotherapy-related side effects and the self-care behaviors of women living with breast cancer: a randomized controlled trial. BMC Nurs. 2023;22(1): 102. 37024875 10.1186/s12912-023-01243-7 PMC10077642

[7] Arnold M, Morgan E, Rumgay H, et al. Current and future burden of breast cancer: global statistics for 2020 and 2040. Breast. 2022;66:15–23. 36084384 10.1016/j.breast.2022.08.010 PMC9465273

[8] Abdel-Razeq H, Mansour A, Jaddan D. Breast Cancer care in Jordan. JCO Glob Oncol. 2020;6260–8. 10.1200/JGO.19.00279. 10.1200/JGO.19.00279 PMC7051801 32083950

[9] Omar S, Khaled H, Gaafar R, Zekry AR, Eissa S, El Khatib O. Breast cancer in Egypt: a review of disease presentation and detection strategies. EMJH- East Mediterr Health J. 2003;9(3):448–63. 15751939

[10] Dey S, Soliman AS, Hablas A, et al. Urban–rural differences in breast cancer incidence by hormone receptor status across 6 years in Egypt. Breast Cancer Res Treat. 2010;120(1):149–60. 10.1007/s10549-009-0427-9. 19548084 10.1007/s10549-009-0427-9 PMC2808467

[11] Arkoob K, Al Nsour M, Al Nemry O, Al Hajawi B. Epidemiology of breast cancer in women in jordan: patient characteristics and survival analysis. EMHJ - East Mediterr Health J. 2010;16(10):1032–8. https://apps.who.int/iris/handle/10665/117999. Published online 2010. Accessed July 21, 2023. 21222418

[12] Akram M, Iqbal M, Daniyal M, Khan AU. Awareness and current knowledge of breast cancer. Biol Res. 2017;50(1):33. 10.1186/s40659-017-0140-9. 28969709 10.1186/s40659-017-0140-9 PMC5625777

[13] Madubogwu CI, Egwuonwu AO, Madubogwu NU, Njelita IA. Breast cancer screening practices amongst female tertiary health worker in Nnewi. J Cancer Res Ther. 2017;13(2):268–75. 10.4103/0973-1482.188433. 28643746 10.4103/0973-1482.188433

[14] Sambanje MN, Mafuvadze B. Breast cancer knowledge and awareness among university students in Angola. Pan Afr Med J. 2012;11(1). 10.4314/pamj.v11i1. PMC3361208 22655104

[15] Hallal JC. The relationship of health beliefs, health locus of control, and self concept to the practice of breast self-examination in adult women. Nurs Res. 1982;31(3):137–42. 6918918

[16] Leung J, McKenzie S, Martin J, Dobson A, McLaughlin D. Longitudinal patterns of breast Cancer screening: mammography, clinical, and breast Self-Examinations in a rural and urban setting. Womens Health Issues. 2014;24(1):e139–46. 10.1016/j.whi.2013.11.005. 24439940 10.1016/j.whi.2013.11.005

[17] Nde FP, Assob JCN, Kwenti TE, Njunda AL, Tainenbe TRG. Knowledge, attitude and practice of breast self-examination among female undergraduate students in the university of Buea. BMC Res Notes. 2015;8(1):43. 10.1186/s13104-015-1004-4. 25889644 10.1186/s13104-015-1004-4 PMC4414436

[18] Villarreal-Garza C, García-Aceituno L, Villa AR, Perfecto-Arroyo M, Rojas-Flores M, León-Rodríguez E. Knowledge about cancer screening among medical students and internal medicine residents in Mexico City. J Cancer Educ. 2010;25(4):624–31. 10.1007/s13187-010-0098-6. 20221811 10.1007/s13187-010-0098-6

[19] Kan’an A. Evaluation of breast cancer (BC) awareness among female university students in Zarqa university, Jordan. Eur J Breast Health. 2018;14(4):199. 30288493 10.5152/ejbh.2018.4048 PMC6170026

[20] Sedrak AS, Galal YS, Amin TT. Cancer screening knowledge and attitudes of under-and post-graduate students at Kasr al Ainy school of medicine, Cairo university, Egypt. Asian Pac J Cancer Prev. 2016;17(8):3809–16. 27644621

[21] Frech A. Healthy behavior trajectories between adolescence and young adulthood. Adv Life Course Res. 2012;17(2):59–68. 10.1016/j.alcr.2012.01.003. 22745923 10.1016/j.alcr.2012.01.003 PMC3381431

[22] Koivusilta LK, Acacio-Claro PJ, Mattila VM, Rimpelä AH. Health and health behaviours in adolescence as predictors of education and socioeconomic status in adulthood – a longitudinal study. BMC Public Health. 2024;24(1):1178. 10.1186/s12889-024-18668-7. 38671433 10.1186/s12889-024-18668-7 PMC11055384

[23] Najjar H, Easson A. Age at diagnosis of breast cancer in Arab nations. Int J Surg. 2010;8(6):448–52. 10.1016/j.ijsu.2010.05.012. 20601253 10.1016/j.ijsu.2010.05.012

[24] Hendrick RE, Monticciolo DL, Biggs KW, Malak SF. Age distributions of breast cancer diagnosis and mortality by race and ethnicity in US women. Cancer. 2021;127(23):4384–92. 10.1002/cncr.33846. 34427920 10.1002/cncr.33846

[25] Zhang QN, Lu HX. Knowledge, attitude, practice and factors that influence the awareness of college students with regards to breast cancer. World J Clin Cases. 2022;10(2):538–46. 10.12998/wjcc.v10.i2.538. 35097079 10.12998/wjcc.v10.i2.538 PMC8771386

[26] Anwar MM, Khalil DM. Breast cancer knowledge, attitude and practice among medical and non-medical university students. J Public Health. 2021;29(4):871–8. 10.1007/s10389-020-01197-z.

[27] Asmare K, Birhanu Y, Wako Z. Knowledge, attitude, practice towards breast self-examination and associated factors among women in Gondar town, Northwest ethiopia, 2021: a community-based study. BMC Womens Health. 2022;22(1):174. 10.1186/s12905-022-01764-4. 35568846 10.1186/s12905-022-01764-4 PMC9107683

[28] Kandasamy G, Almaghaslah D, Almanasef M, Alamri RDA. Knowledge, attitude, and practice towards breast self-examination among women: a web based community study. Front Public Health. 2024;12:1450082. 10.3389/fpubh.2024.1450082. 39435407 10.3389/fpubh.2024.1450082 PMC11491359

[29] Jadhav BN, Abdul Azeez EP, Mathew M, et al. Knowledge, attitude, and practice of breast self-examination is associated with general self-care and cultural factors: a study from Tamil nadu, India. BMC Womens Health. 2024;24(1):151. 10.1186/s12905-024-02981-9. 38431649 10.1186/s12905-024-02981-9 PMC10909289

[30] Muhsin M, Mohamad Yamin LS. Knowledge, attitude, and practice regarding breast Self-Examination among female health sciences final year students. Malays J Med Health Sci. 2022;18. 10.47836/mjmhs18.s15.28.

[31] Kharaba Z, Buabeid MA, Ramadan A, et al. Knowledge, attitudes, and practices concerning breast Cancer and self examination among females in UAE. J Community Health. 2021;46(5):942–50. 10.1007/s10900-021-00969-2. 33754294 10.1007/s10900-021-00969-2

[32] von Elm E. STROBE initiative. The strengthening the reporting of observational studies in epidemiology (STROBE) statement: guidelines for reporting observational studies. J Clin Epidemiol. 2008;61:344. 18313558 10.1016/j.jclinepi.2007.11.008

[33] Dean AG. OpenEpi: open source epidemiologic statistics for public health, version 2.3. 1. *Httpwww Openepi Com*. Published online 2010.

[34] Naing L, Nordin RB, Abdul Rahman H, Naing YT. Sample size calculation for prevalence studies using scalex and scalar calculators. BMC Med Res Methodol. 2022;22(1):209. 10.1186/s12874-022-01694-7. 35907796 10.1186/s12874-022-01694-7 PMC9338613

[35] Boulos DN, Ghali RR. Awareness of breast cancer among female students at Ain Shams university, Egypt. Glob J Health Sci. 2013;6(1):154. 24373275 10.5539/gjhs.v6n1p154 PMC4825266

[36] Omar A, Bakr A, Ibrahim N. Female medical students’ awareness, attitudes, and knowledge about early detection of breast cancer in Syrian private university, Syria. Heliyon. 2020. 10.1016/j.heliyon.2020.e03819. 32368654 10.1016/j.heliyon.2020.e03819 PMC7184173

[37] Osei-Afriyie S, Addae AK, Oppong S, Amu H, Ampofo E, Osei E. Breast cancer awareness, risk factors and screening practices among future health professionals in Ghana: a cross-sectional study. PLoS One. 2021;16(6): e0253373. 10.1371/journal.pone.0253373. 34166407 10.1371/journal.pone.0253373 PMC8224936

[38] Hussain I, Majeed A, Masood I, et al. A national survey to assess breast cancer awareness among the female university students of Pakistan. Soofi SB, ed *PLOS ONE*. 2022;17(1):e0262030. 10.1371/journal.pone.0262030 PMC8782286 35061770

[39] Mehiret G, Molla A, Tesfaw A. Knowledge on risk factors and practice of early detection methods of breast cancer among graduating students of Debre Tabor university, northcentral Ethiopia. BMC Womens Health. 2022;22(1):183. 35585540 10.1186/s12905-022-01768-0 PMC9118614

[40] Koh KL, Ahad NA. Normality for non-normal distributions. J Sci Math Lett. 2020;8(2):51–60. 10.37134/jsml.vol8.2.7.2020.

[41] El Saghir NS, Khalil MK, Eid T, et al. Trends in epidemiology and management of breast cancer in developing Arab countries: a literature and registry analysis. Int J Surg. 2007;5(4):225–33. 10.1016/j.ijsu.2006.06.015. 17660128 10.1016/j.ijsu.2006.06.015

[42] Tarabeia J, Baron-Epel O, Barchana M, et al. A comparison of trends in incidence and mortality rates of breast cancer, incidence to mortality ratio and stage at diagnosis between Arab and Jewish women in Israel, 1979–2002. Eur J Cancer Prev. 2007;16(1):36–42. 10.1097/01.cej.0000228407.91223.85. 17220702 10.1097/01.cej.0000228407.91223.85

[43] Donnelly TT, Al Khater AH, Al-Bader SB, et al. Arab women’s breast cancer screening practices: a literature review. Asian Pac J Cancer Prev. 2013;14(8):4519–28. 10.7314/APJCP.2013.14.8.4519. 24083695 10.7314/apjcp.2013.14.8.4519

[44] Chouchane L, Boussen H, Sastry KSR. Breast cancer in Arab populations: molecular characteristics and disease management implications. Lancet Oncol. 2013;14(10):e417–24. 10.1016/S1470-2045(13)70165-7. 23993386 10.1016/S1470-2045(13)70165-7

[45] Petroc-Nustas WI. Factors associated with mammography utilization among Jordanian women. J Transcult Nurs. 2001;12(4):284–91. 10.1177/104365960101200403. 11989219 10.1177/104365960101200403

[46] Nemenqani DM, Abdelmaqsoud SH, Al-Malki AHA, Oraija AA, Al-Otaibi EM. Knowledge, attitude and practice of breast self examination and breast cancer among female medical students in Taif, Saudi Arabia. Open J Prev Med. 2014;04(02):69–77. 10.4236/ojpm.2014.42011.

[47] Donnelly TT, Al Khater AH, Al-Bader SB, et al. Factors that influence awareness of breast cancer screening among Arab women in Qatar: results from a cross sectional survey. Asian Pac J Cancer Prev. 2015;15(23):10157–64. 10.7314/APJCP.2014.15.23.10157. 10.7314/apjcp.2014.15.23.10157 25556441

[48] Petro-Nustus W, Mikhail BI. Factors associated with breast self‐examination among Jordanian women. Public Health Nurs. 2002;19(4):263–71. 10.1046/j.1525-1446.2002.19406.x. 12071900 10.1046/j.1525-1446.2002.19406.x

[49] Suleiman A. Awareness and attitudes regarding breast cancer and breast self-examination among female Jordanian students. J Basic Clin Pharm. 2014;5(3):74. 10.4103/0976-0105.139730. 25278670 10.4103/0976-0105.139730 PMC4160723

[50] Lera T, Beyene A, Bekele B, Abreha S. Breast self-examination and associated factors among women in Wolaita sodo, ethiopia: a community-based cross-sectional study. BMC Womens Health. 2020;20(1):167. 10.1186/s12905-020-01042-1. 32770978 10.1186/s12905-020-01042-1 PMC7414537

[51] Getu MA, Abebe M, Tlaye KG, Goshu AT. Breast Self-Examination knowledge and its determinants among female students at addis Ababa university, ethiopia: an Institution‐Based Cross‐Sectional study. Fancellu A. Ed BioMed Res Int. 2022;2022(1):2870419. 10.1155/2022/2870419. 10.1155/2022/2870419 PMC9170413 35677100

[52] Sahloul E, Salem R, Alrez W, et al. Cancer care at times of crisis and war: the Syrian example. J Glob Oncol. 2017;3(4):338–45. 10.1200/JGO.2016.006189. 28831442 10.1200/JGO.2016.006189 PMC5560458

[53] Mansour R, Abdel-Razeq H, Al-Hussaini M, et al. Systemic barriers to optimal Cancer care in Resource-Limited countries: Jordanian healthcare as an example. Cancers. 2024;16(6):1117. 10.3390/cancers16061117. 38539452 10.3390/cancers16061117 PMC10968872

[54] Khader Y, Al Nsour M, Abu Khudair S, Saad R, Tarawneh MR, Lami F. Strengthening primary healthcare in Jordan for achieving universal health coverage: A need for family health team approach. Healthcare. 2023;11(22):2993. 10.3390/healthcare11222993. 37998485 10.3390/healthcare11222993 PMC10671215

[55] Hammad EA. The use of economic evidence to inform drug pricing decisions in Jordan. Value Health. 2016;19(2):233–8. 10.1016/j.jval.2015.11.007. 27021758 10.1016/j.jval.2015.11.007

[56] Rihani R, Jeha S, Nababteh M, Rodriguez-Galindo C, Mansour A, Sultan I. The burden and scope of childhood cancer in displaced patients in Jordan: the King Hussein Cancer center and foundation experience. Front Oncol. 2023;13: 1112788. 10.3389/fonc.2023.1112788. 37035175 10.3389/fonc.2023.1112788 PMC10080160

[57] Nazer LH, Tuffaha H. Health care and pharmacy practice in Jordan. Can J Hosp Pharm. 2017;70(2). 10.4212/cjhp.v70i2.1649. 10.4212/cjhp.v70i2.1649 PMC5407425 28487583

[58] Qtaishat E, Al-Ajlouni R, Ammar K, et al. Exploring barriers to early breast examination and screening among Arab women in the MENA region: A KAP study. Heliyon. 2025;11(3):e42167. 10.1016/j.heliyon.2025.e42167. 39944346 10.1016/j.heliyon.2025.e42167 PMC11815657

[59] Eysenbach G. Improving the quality of web surveys: the checklist for reporting results of internet E-Surveys (CHERRIES). J Med Internet Res. 2004;6(3):e34. 10.2196/jmir.6.3.e34. 15471760 10.2196/jmir.6.3.e34 PMC1550605

