# Supplementary material for: Knowledge, attitudes, and awareness of breast self-examination among female university students in three Arab countries: a multi-center cross-sectional study
Source: BMC Public Health. 2025 Aug 27;25:2961. doi: 10.1186/s12889-025-23932-5 (PMC12382118; doi:10.1186/s12889-025-23932-5)
Supplement: Supplementary file 1 — Supplementary Material 1. [file 12889_2025_23932_MOESM1_ESM.docx]

**Knowledge, Attitudes, and Awareness of Early Breast Cancer Detection Among Female University Students in Three Arab Countries: A Multi-center Cross-sectional Study**

**Short running title: Breast Cancer in Arab Countries**

Aliaa Gamal^1^, Sara Adel Awwad^2^, Waleed Alhayek^3^, Hagar Elgamal^4^, Sarah Saleh Omar^5^, Abdallfatah Abdallfatah^6^, Noura Waleed Koura^7^, Abdelrahman Sameh Soliman^8^, Abdelazeez Ahmed Masoud^9^, Obai Yousef ^10^, Yasmeen Jamal Alabdallat^11^, Mohamed Abouzid^12,13^

**Affiliations**

1. Clinical Pharmacy Department, Faculty of Pharmacy, Deraya University, Minia, Egypt; Email: aliaagamal2622@gmail.com
2. Faculty of Medicine, Jordan University of Science and Technology, Irbid, Jordan; Email: sarahawwad74@gmail.com
3. Tishreen University, Faculty of Medicine, Lattakia, Syria; Email: waledalhaek9@gmail.com
4. Public Administration of Dental Health, Health Affairs Directorate Alexandria, Egypt; Email: Hagar.elgamal2611@gmail.com
5. Faculty of Medicine and Health Sciences, Aden University, Aden, Yemen; Email: sarahsalehomar@gmail.com
6. Faculty of Medicine, October 6 University, Egypt; Email: Abdallfatahofficial@gmail.com
7. Faulty of Medicine, Kafr El-sheik University, Kafr El-sheik, Egypt; Email: nourawaleed611@gmail.com
8. Faulty of Medicine, Alexandria University, Alexandria, Egypt; Email: abdelrahmansameh59@yahoo.com
9. Faulty of Medicine, Alexandria University, Alexandria, Egypt; Email: abdelazeez.ahmed12@gmail.com
10. Faculty of Medicine, Tartous University, Syria; Email: obai.mahmoud.yousef@gmail.com
11. Faculty of Medicine, Hashemite University, 13133, Zarqa, Jordan; Email: [abdallat.01@gmail.com](mailto:abdallat.01@gmail.com)
12. Department of Physical Pharmacy and Pharmacokinetics, Faculty of Pharmacy, Poznan University of Medical Sciences, Rokietnicka 3 St., 60-806, Poznan, Poland ([mmahmoud@ump.edu.pl](mailto:mmahmoud@ump.edu.pl)).
13. Doctoral School, Poznan University of Medical Sciences, Poznan, 60-812, Poland

Authors' ORCID IDs **[
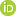
](about:blank)**

Aliaa Gamal: [**https://orcid.org/0000-0001-7508-4120**](https://orcid.org/0000-0001-7508-4120%20)

Sara Adel Awwad: 0000-0001-9159-2731

Waleed Alhayek: 0009-0004-1864-4629

Hagar Elgamal: 0000-0003-2236-1272

Sarah Saleh Omar: 0009-0002-8002-6923

Abdallfatah Abdallfatah: 0009-0007-2055-9134

Noura Waleed koura: 0009-0008-8156-5180

Abdelrahman Sameh Soliman: 0009-0004-3944-7712

Abdelazeez Ahmed Masoud: 0009-0007-7267-1961

Obai Yousef: <https://orcid.org/0009-0001-5832-3780>

*Yasmeen Jamal Alabdallat*: 0000-0001-6855-3718.

Mohamed Abouzid: <https://orcid.org/0000-0002-8917-671X>

**Section 1: Demographics**

1 ) Age:

2) Academic Field

A. Health care related fields (Medicine, dentistry, pharmacy …etc)

B. Non-health care related fields

3) Academic year

A. First year

B. Second year

C. Third year

D. Fourth year

E. Fifth year

F. Sixth year

G. Internship year

4) Mother’s Education

A. Less than high school

B. High school diploma

C. Bachelor’s degree

D. Master’s degree.

E. PhD

5) Place of residency

A. Jordan

B. Egypt

C. Syria

D. Other

7) Marital Status:

A. Married

B. Engaged

C. Singled

8) Family History of Breast Cancer:

A. Yes

B. No

C. Do not know

**Section 2: Knowledge and awareness of breast cancer: (Yes/No/Don’t know)**

1. Which of the following are risk factors of breast cancer?

1) Early puberty/menarche (Less than 12 years) Yes

2) Late menopause (after the age of 55) Yes

3) Oral contraceptives Yes

4) Postmenopausal estrogens Yes

5) History of trauma on the breast Yes

6) Having a past history of benign tumors Yes

7) History of inflammatory disease of the breast Yes

8) Alcohol Yes

9) Smoking Yes

10) Family history of breast cancer Yes

11) Not being physically active Yes

12) Being obese / obesity Yes

13) Aging Yes

14) High fat diet Yes

15) Genetic Factors Yes

16) Never having been pregnant Yes

17) Having a past history of breast cancer Yes

18) Never breast fed Yes

19) Interacting with patients with breast cancer No

2. Which of the following are common clinical features of breast cancer?

1. A lump or thickening in the breast Yes
2. A lump or thickening under the armpit Yes
3. Changes in pigmentation Yes
4. Bloody nipple discharge (other than breast milk) Yes
5. Change in nipple position Yes
6. Change in the shape of the breast or the nipple (e.g. nipple retraction… etc) Yes
7. Change in the size of the breast or the nipple Yes
8. Breast Asymmetry Yes
9. Redness of the nipple or breast skin Yes
10. Skin rash on the breast Yes
11. Pain in one of the breasts or the armpit Yes

3. What is your source of information on breast cancer?

A. Health care professionals

B. Awareness campaigns in universities

C. Books

D. Television

E. Relatives/Friends

F. Social media

**Section 2: Knowledge on Early Detection Measures:**

The following measures are used for the early detection of breast cancer.

1. BSE (True)
2. Mammogram (True)
3. Ultra-sound (True)

**Section 3: Knowledge and Practice of Breast Self-Examination:**

*Knowledge of BSE*

1. Have you heard before about BSE? (Yes/No)

**If the answer to the previous question is no, the form is submitted.**

2. How often do you think BSE should be performed?

A. Daily

B. Weekly

C. Monthly (True)

D. Yearly

E. I don’t know

3. When do you think is the right time for a woman to perform BSE?

A. Before menstruation

B. Middle of menstruation (day 3–5)

C. Any day during menstruation

D. After menstruation (True)

E. Anytime

F. Don’t know

*Practice of BSE:*

4. Do you perform BSE?

Yes (1 point)/No(Zero)

**If your answer is No, leave Q6 to Q10 unanswered. If it is yes leave Q5 unanswered and keep going.**

5. If No, why?

A. Fear of positive finding (Zero)

B. Forgetting (Zero)

C. Not sure of its ability to detect breast cancer (Zero)

D. Don’t know how (Zero)

E. Not interested (Zero)

6. If yes, how often do you check your breasts?

A. Rarely (Zero)

B. At least once every month (3 points)

C. At least once every 6 months (2 points)

D. At least once a year (1 point)

7. How confident are you that you would notice a change in your breasts?

A. Not confident at all (Zero)

B. Not very confident (1 points)

C. Fairly confident (2 points)

D. Very confident (3 points)

8. Would you go to see a doctor to check your breast if you were **very** confident about detecting a change in your breast? (Yes(1 point)/No(Zero)/Don’t know(Zero))

9. Would you go to see a doctor to check your breast if you were **fairly** confident about detecting a change in your breast? (Yes(2 points)/No(Zero)/Don’t know(Zero))

**If you answered any of the previous two questions with no or don’t know, proceed to the next questions. If you didn’t, just leave them unanswered.**

Would you tell us how the factors below may make you refrain from doing a clinical examination of your breast or hesitant about the idea?

10. Fear of positive result

A. Strongly disagree (2 points)

B. Disagree (1 point)

C. Neuter(Zero)

D. Agree(Zero)

E. Strongly Agree(Zero)

11 Shyness

A. Strongly disagree(2 points)

B. Disagree (1 point)

C. Neuter(Zero)

D. Agree(Zero)

E. Strongly Agree(Zero)

12. Don’t want to panic your parents

A. Strongly disagree (2 points)

B. Disagree (1 point)

C. Neuter(Zero)

D. Agree(Zero)

E. Strongly Agree (Zero)

13. You don’t want to spend money on a test that you are not sure is necessary for you

A. Strongly disagree (2 points)

B. Disagree (1 point)

C. Neuter (Zero)

D. Agree (Zero)

E. Strongly Agree (Zero)

**References:**

[1.    Alsowiyan AA, Almotyri HM, Alolayan NS, Alissa LI, Almotyri BH, AlSaigh SH. Breast cancer knowledge and awareness among females in Al-Qassim Region, Saudi Arabia in 2018. J Family Med Prim Care. 2020 Mar 26;9(3):1712–8.](https://sciwheel.com/work/bibliography/14312884)

[2.    Akter MF, Ullah MO. Awareness levels of breast cancer among female university and medical college students in Sylhet city of Bangladesh. Cancer Rep (Hoboken). 2022 Nov;5(11):e1608.](https://sciwheel.com/work/bibliography/12910105)

[3.    Ismail H, Shibani M, Zahrawi HW, Slitin AF, Alzabibi MA, Mohsen F, et al. Knowledge of breast cancer among medical students in Syrian Private University, Syria: a cross-sectional study. BMC Med Educ. 2021 May 1;21(1):251.](https://sciwheel.com/work/bibliography/14312033)

[4.    Rahman SA, Al-Marzouki A, Otim M, Khalil Khayat NEH, Yousuf R, Rahman P. Awareness about Breast Cancer and Breast Self-Examination among Female Students at the University of Sharjah: A Cross-Sectional Study. Asian Pac J Cancer Prev. 2019 Jun 1;20(6):1901–8.](https://sciwheel.com/work/bibliography/14220503)

5. Boulos DN, Ghali RR. Awareness of breast cancer among female students at Ain

Shams University, Egypt. Glob J Health Sci. 2013 Nov 4;6(1):154-61. doi:

10.5539/gjhs.v6n1p154. PMID: 24373275; PMCID: PMC4825266.
