# Supplementary material for: Knowledge, attitudes, and awareness of breast self-examination among female university students in three Arab countries: a multi-center cross-sectional study
Source: BMC Public Health. 2025 Aug 27;25:2961. doi: 10.1186/s12889-025-23932-5 (PMC12382118; doi:10.1186/s12889-025-23932-5)
Supplement: Supplementary file 2 — Supplementary Material 2. [file 12889_2025_23932_MOESM2_ESM.docx]

| Table S1. Main Source of Information on Breast Cancer | | | | |
| --- | --- | --- | --- | --- |
|  |  | Academic Field | |  |
| What is your main source of information on breast cancer? |  | Healthcare related fields | Non-health care related fields | Total |
| Awareness campaigns Uni | Observed | 121 | 25 | 146 |
|  | % within column | 12.38 | 6.51 | 10.73 |
| Books | Observed | 210 | 17 | 227 |
|  | % within column | 21.49 | 4.43 | 16.68 |
| Health care professionals | Observed | 267 | 29 | 296 |
|  | % within column | 27.33 | 7.55 | 21.75 |
| Relatives / friends | Observed | 67 | 65 | 132 |
|  | % within column | 6.86 | 16.93 | 9.7 |
| Social media | Observed | 287 | 222 | 509 |
|  | % within column | 29.38 | 57.81 | 37.4 |
| Television | Observed | 25 | 26 | 51 |
|  | % within column | 2.56 | 6.77 | 3.75 |
| Total | Observed | 977 | 384 | 1361 |
|  | % within column | 100.0 % | 100.0 % | 100.0 % |
| *P* value of Chi square Test | **< 0.001** |  |  |  |

| Table S2. Risk Factors | | | | | |
| --- | --- | --- | --- | --- | --- |
| Breast Cancer History (benign or malignant), and Medication | | | | | |
| Risk factor |  | Overall | Place of residency  N (%) | | |
|  |  | N (%) | Egypt | Jordan | Syria |
| History of trauma on the breast | TRUE | 605 (44%) | 212 (49.19) | 212 (49.30) | 181 (36.20) |
|  | FALSE | 431 (32%) | 133 (30.86) | 107 (24.88) | 191 (38.20) |
|  | Don’t know | 325 (24%) | 86 (19.95) | 111 (25.81) | 128 (25.60) |
| History of inflammatory disease of the breast | TRUE | 942 (69%) | 300 (69.61) | 314 (73.02) | 328 (65.60) |
|  | FALSE | 185 (14%) | 62 (14.39) | 49 (11.40) | 74 (14.80) |
|  | Don’t know | 234 (17%) | 69 (16.01) | 67 (15.58) | 98 (19.60) |
| Having a history of benign tumors | TRUE | 1,005 (74%) | 329 (76.33) | 329 (76.51) | 347 (69.40) |
|  | FALSE | 175 (13%) | 56 (12.99) | 48 (11.16) | 71 (14.20) |
|  | Don’t know | 181 (13%) | 46 (10.67) | 53 (12.33) | 82 (16.40) |
| Personal History of BC | TRUE | 1,245 (91%) | 395 (91.65) | 387 (90.00) | 463 (92.60) |
|  | FALSE | 59 (4.3%) | 22 (5.10) | 20 (4.65) | 17 (3.40) |
|  | Don’t know | 57 (4.2%) | 14 (3.25) | 23 (5.35) | 20 (4.00) |
| Family history of BC | TRUE | 1,239 (91%) | 395 (91.65) | 383 (89.07) | 461 (92.20) |
|  | FALSE | 65 (4.8%) | 20 (4.64) | 21 (4.88) | 24 (4.80) |
|  | Don’t know | 57 (4.2%) | 16 (3.71) | 26 (6.05) | 15 (3.00) |
| Oral contraceptives | TRUE | 716 (53%) | 265 (61.48) | 223 (51.86) | 228 (45.60) |
|  | FALSE | 275 (20%) | 62 (14.39) | 76 (17.67) | 137 (27.40) |
|  | Don’t know | 370 (27%) | 104 (24.13) | 131 (30.47) | 135 (27.00) |
| Postmenopausal estrogens | TRUE | 754 (55%) | 265 (61.48) | 218 (50.70) | 271 (54.20) |
|  | FALSE | 175 (13%) | 57 (13.23) | 55 (12.79) | 63 (12.60) |
|  | Don’t know | 432 (32%) | 109 (25.29) | 157 (36.51) | 166 (33.20) |
| Women’s Health Issues | | | | | |
| Early puberty/menarche (Less than 12 years) | TRUE | 449 (33%) | 134 (31.09) | 141 (32.79) | 174 (34.80) |
|  | FALSE | 537 (39%) | 187 (43%) | 153 (36%) | 197 (39%) |
|  | Don’t know | 375 (28%) | 187 (43.39) | 153 (35.58) | 197 (39.40) |
| Late menopause (after the age of 55) | TRUE | 551 (40%) | 220 (51%) | 157 (37%) | 174 (35%) |
|  | FALSE | 420 (31%) | 110 (25.52) | 136 (31.63) | 129 (25.80) |
|  | Don’t know | 390 (29%) | 220 (51.04) | 157 (36.51) | 174 (34.80) |
| Never having been pregnant | TRUE | 505 (37%) | 172 (40%) | 150 (35%) | 183 (37%) |
|  | FALSE | 466 (34%) | 111 (25.75) | 121 (28.14) | 188 (37.60) |
|  | Don’t know | 390 (29%) | 100 (23.20) | 152 (35.35) | 138 (27.60) |
| Never breast fed | TRUE | 526 (39%) | 172 (39.91) | 150 (35%) | 183 (37%) |
|  | FALSE | 388 (29%) | 119 (28%) | 115 (27%) | 154 (31%) |
|  | Don’t know | 447 (33%) | 164 (38.05) | 131 (30.47) | 171 (34.20) |
| Genetic Factors | TRUE | 553 (40.6%) | 220 (51.04) | 150 (34.88) | 183 (36.6) |
|  | FALSE | 538 (39.5%) | 187 (43.38) | 157 (34.65) | 194 (38.8) |
|  | Don’t know | 270 (19.9%) | 100 (23.2%) | 120 (27.9%) | 50 (10%) |
| Lifestyle and Physical Activity | | | | | |
| Risk factor |  | Overall | Place of residency  N (%) |  |  |
|  |  | N (%) | Egypt | Jordan | Syria |
| Alcohol | TRUE | 799 (59%) | 262 (60.79) | 264 (61.40) | 273 (54.60) |
|  | FALSE | 210 (15%) | 71 (16.47) | 50 (11.63) | 89 (17.80) |
|  | Don’t know | 352 (26%) | 98 (22.74) | 116 (26.98) | 138 (27.60) |
| Smoking | TRUE | 893 (66%) | 288 (66.82) | 280 (65.12) | 325 (65.00) |
|  | FALSE | 199 (15%) | 72 (16.71) | 55 (12.79) | 72 (14.40) |
|  | Don’t know | 269 (20%) | 71 (16.47) | 95 (22.09) | 103 (20.60) |
| Not being physically active | TRUE | 526 (39%) | 144 (33.41) | 185 (43.02) | 197 (39.40) |
|  | FALSE | 405 (30%) | 154 (35.73) | 112 (26.05) | 139 (27.80) |
|  | Don’t know | 430 (32%) | 133 (30.86) | 133 (30.93) | 164 (32.80) |
| Being obese / obesity | TRUE | 694 (51%) | 225 (52.20) | 220 (51.16) | 249 (49.80) |
|  | FALSE | 328 (24%) | 110 (25.52) | 97 (22.56) | 121 (24.20) |
|  | Don’t know | 339 (25%) | 96 (22.27) | 113 (26.28) | 130 (26.00) |
| Aging | TRUE | 730 (54%) | 232 (53.83) | 236 (54.88) | 262 (52.40) |
|  | FALSE | 360 (26%) | 117 (27.15) | 101 (23.49) | 142 (28.40) |
|  | Don’t know | 271 (20%) | 82 (19.03) | 93 (21.63) | 96 (19.20) |

**Supplementary Figure 1. Source of information of BC and place of residency**
